# Ribavirin suppresses bacterial virulence by targeting LysR-type transcriptional regulators

**DOI:** 10.1038/srep39454

**Published:** 2016-12-19

**Authors:** Rahul Shubhra Mandal, Atri Ta, Ritam Sinha, Nagaraja Theeya, Anirban Ghosh, Mohsina Tasneem, Anirban Bhunia, Hemanta Koley, Santasabuj Das

**Affiliations:** 1 Biomedical Informatics Center, National Institute of Cholera and Enteric Diseases, Kolkata, India; 2 Division of Clinical Medicine, National Institute of Cholera and Enteric Diseases, Kolkata, India; 3 Division of Bacteriology, National Institute of Cholera and Enteric Diseases, Kolkata, India; 4 Department of Biophysics, Bose Institute, Kolkata, India

## Abstract

Targeting bacterial virulence mechanisms without compromising bacterial growth is a promising strategy to prevent drug resistance. LysR-type transcriptional regulators (LTTRs) possess structural conservation across bacterial species and regulate virulence in numerous pathogens, making them attractive targets for antimicrobial agents. We targeted AphB, a *Vibrio cholerae* LTTR, which regulates the expression of genes encoding cholera toxin and toxin-co-regulated pilus for inhibitor designing. Since AphB ligand is unknown, we followed a molecular fragment-based approach for ligand designing using FDA-approved drugs and subsequent screen to identify molecules that exhibited high-affinity binding to AphB ligand-binding pocket. Among the identified compounds, ribavirin, an anti-viral drug, antagonized AphB functions. Ribavirin perturbed *Vibrio cholerae* pathogenesis in animal models. The inhibitory effects of the drug was limited to the bacteria expressing wild type AphB, but not its constitutively active mutant (AphB_N100E_), which represents the ligand-bound state, suggesting that ribavirin binds to the active site of AphB to exert its inhibitory role and there exists no AphB-independent mechanism of its action. Similarly, ribavirin suppressed the functions of *Salmonella* Typhi LTTR Hrg, indicating its broad spectrum efficacy. Moreover, ribavirin did not affect the bacterial viability in culture. This study cites an example of drug repurposing for anti-infective therapy.

Antibiotic resistance of pathogenic bacteria has become a major public health threat worldwide^1^. Drug-resistant pathogens not only increase the morbidity and mortality, but also multiply the treatment costs by several folds^2^. The development and rapid spread of multi-drug resistant (MDR) strains, especially among the Gram negative enterobacteriaceae have emerged from the widespread use of antibiotics, often inappropriately and in sub-therapeutic doses. The strong evolutionary pressure of cell death due to the use of antibiotics gives significant survival advantage to the bacteria carrying resistant mutations^3^, which subsequently spread to other bacterial species as horizontally acquired elements. The threat imposed by antibiotic-resistant pathogens is further magnified by the availability of fewer novel compounds to treat bacterial infections^4^,^5^. The prevailing situation has motivated the scientists to explore new strategies for antibacterial drug discovery.

Anti-virulence strategies are particularly attractive, because they may be highly effective in the treatment of bacterial infections, while minimizing drug resistance. Drugs that specifically target the virulence mechanisms, such as adhesion/invasion of the host cells, biofilm formation, toxin production, virulence gene expression and secretion of virulence factors etc. will inhibit pathogenesis without compromising growth or survival of the organisms^6^. Chemical inhibitors blocking toxins, pilins, quorum sensing molecules, transcriptional regulators of virulence genes, type three secretion systems and histidine kinases have been reported in the literature^6^.

The LysR family proteins are global transcriptional regulators (LysR-type transcriptional regulators), widely distributed in the prokaryotes. They constitute a major group of bacterial virulence determinants through the regulation of quorum sensing, motility, oxidative stress responses, toxin production, attachment, secretion etc^7^. Therefore LTTRs can be used as potential targets for anti-bacterial drug development. A *Vibrio cholerae* LTTR called AphB functions as a master regulator of the virulence phenotype. AphB, acting together with AphA drive transcription from the *tcpPH* promoter^8^. The *tcpPH* operon encodes two transmembrane regulatory proteins, TcpP and TcpH, which co-operate with ToxR and ToxS to activate the *toxT* gene, a direct transcriptional activator of the virulence genes, *ctxAB* and *tcpA*^9^*. ctxAB* encodes CT, an enterotoxin responsible for severe diarrhoea during cholera, while TcpA is the major sub-unit of toxin-coregulated pilus (TCP) and is essential for the attachment and colonization of the intestinal epithelium by *Vibrio cholerae*^10^. Recently, the crystal structure of AphB was solved. The N-terminal DNA-binding helix-turn-helix (residues 1–58) and C-terminal co-inducer-binding regulatory (residues 90–291) domains of AphB are connected by a linker helix (residues 59–89). Two extended antiparallel β-strands (β6 and β12) connect the RD-I (residues 90–159 and residues 262–291) and RD-II (residues 160–261) sub domains of the regulatory domain^11^.

We performed *in silico* screening of a library constructed from the FDA-approved drug fragments to find molecules that bind AphB at the putative ligand/ co-inducer-binding pocket and joined them to build novel molecular scaffolds. Molecular sub-structure-based screening identified a small molecule compound, ribavirin, which is a clinically approved antiviral drug. Ribavirin interacted with AphB and inhibited its functions, leading to the suppression of CT production and abrogation of *V. cholerae* colonization and pathogenesis in animal models. Ribavirin also inhibited Hrg, an LTTR from *Salmonella enterica* subspecies *enterica* serovar Typhi (*S.* Typhi) and protected mice against systemic infections due to the organism. However, the drug molecule exerted no direct bacteriostatic or bactericidal effects. Thus, ribavirin is a novel therapeutic agent for bacterial infections that functions through substrate-competitive inhibition of LTTRs.

## Results

### Generation of fragment-based molecules

**Figure 1:**
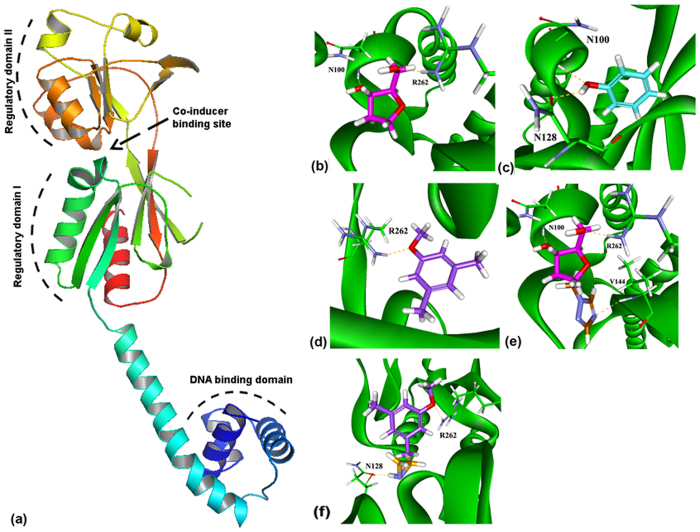
Docked conformation of FDA approved drug fragments. (**a**) Crystal structure of AphB showing co-inducer binding site along with other domains (**b**) MFCD01075954 (pink), (**c**) MFCD00002143 (cyan) and (**d**) MFCD00008398 (violet) show H-bonding with active site critical residues of AphB. Docking was done using LUDI method in Discovery Studio 2.5 software. (**e**) Docked pose of the linked-fragment joining MFCD01075954 and MFCD01076192 by De Novo Link method shows H-bonding with N100, R262 and V144. (**e**) Docked pose of MFCD00008398 joined with the fragment MFCD00039669, showing H-bonding with N128 and R262.

To design molecules that could compete with the putative ligand/co-inducer for binding to the AphB ligand-binding pocket (Fig. 1a), we have used LUDI-based methods available in the Discovery Studio 2.5 software. FDA-approved drugs fragments were prepared for LUDI using De Novo Library Generation method^12^. The fragments with the potential to bind to the critical residues, such as N100, N128, V144, L194 and R262 of the ligand-binding pocket of AphB were screened using LUDI De Novo Receptor in Discovery Studio 2.5. Mutations of these residues were reported earlier to result in the loss of AphB functions or constitutive activation of AphB^11^. Selection of the AphB-interacting fragments was done based on the hydrogen-bonding (H-bonding) with one or more of the above residues. We found three drug fragments with FDA fragment id MFCD01075954, MFCD00002143 and MFCD00008398, which formed H-bond interactions with N100 and R262, N128 and N100, and R262 residues of AphB, respectively (Fig. 1b–e).

To build novel molecular scaffolds, which could accommodate the AphB ligand-binding pocket out of the above three fragments, we employed LUDI De Novo Link method^12^ and designed multiple linked-fragments for each of the above fragments. Further analysis was done with those linked molecules, which made additional H-bonds compared with the individual drug fragments with the critical ligand-binding residues of AphB. Joining MFCD01076192 with MFCD01075954 resulted in additional H-bonding with V144, generating a novel molecular scaffold (Fig. 1e). Another linked-fragment that joined MFCD00039669 and MFCD00008398 formed H-bonds with R262 and N128 (Fig. 1f). However, linking MFCD00002143 with no other drug fragment increased H-bond interactions within the ligand-binding pocket.

### Molecular substructure search and molecular docking

**Figure 2:**
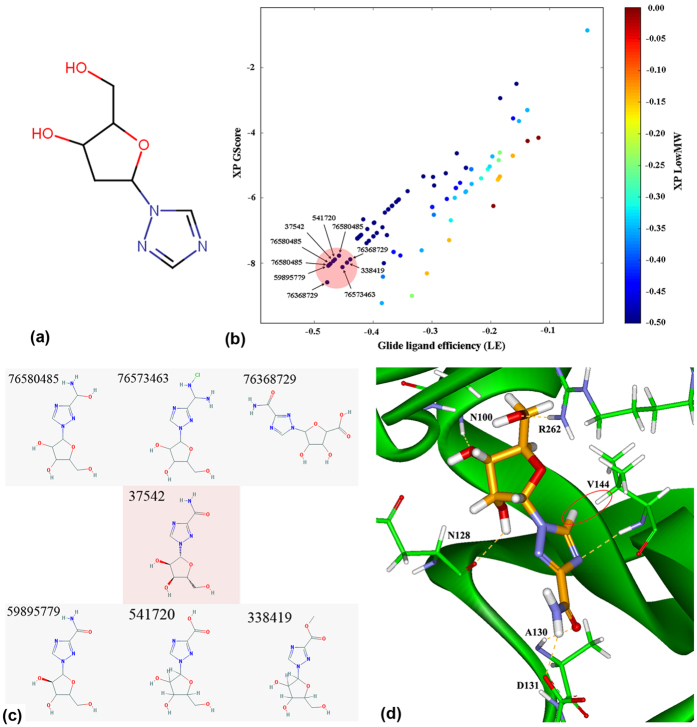
Computational selection of AphB ligand molecule. (**a**) Linked-fragment joining MFCD01075954 and MFCD01076192. (**b**) Ligand efficiency (LE) plot of top 10 poses, labeled as respective PubChem CID and generated by Glide XP^13^. (**c**) Top seven compounds selected as per their respective ligand efficiency (LE). (**d**) Docked conformation of Ribavirin within AphB co-inducer-binding site, showing H-bonding with several critical residues. The red circle denotes the proton found in the near vicinity of V144 by STD-NMR.

**Figure 3:**
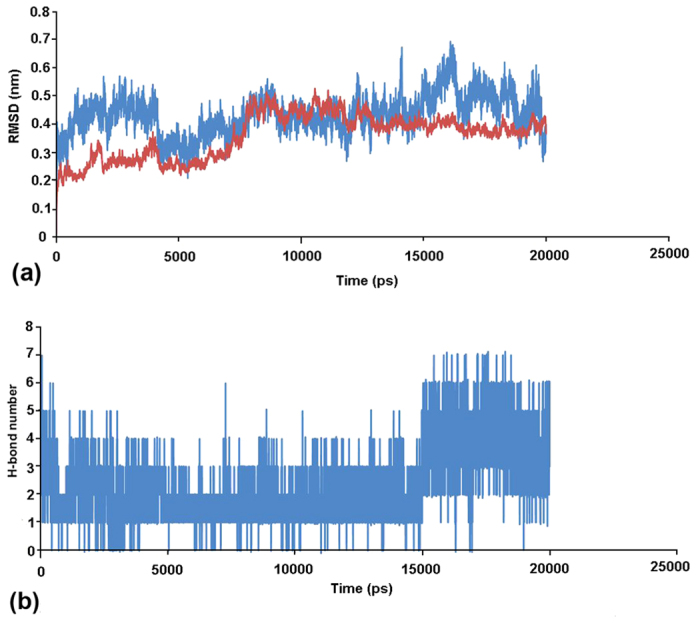
20 ns molecular dynamics simulation plot of AphB-ribavirin complex. (**a**) RMSD plot showing stability of the Ribavirin (Blue)-AphB(Red) backbone conformation. (**b**) Number of H-bond interactions observed between Ribavirin and AphB.

Out of the novel molecular scaffolds generated by linking the FDA-approved drug fragments, we selected molecule joining MFCD01075954 and MFCD01076192 fragments (Fig. 2a) for substructure-based searching in PubChem database. The other joined molecule did not produce any substructure having that scaffold; moreover the selected joined-molecule produced more H-bond interactions with the critical ligand-binding site residues of AphB. The PubChem substructure-based search identified 1087 drug-like small molecules, which were screened by docking to the AphB ligand-binding pocket using GLIDE XP^13^ and 100 best docked poses were selected. Finally, top ten poses (Fig. 2b) of seven unique molecules (Fig. 2c) were selected based on their ligand efficiency (LE). It is calculated as LE = (ΔG)/N, where ΔG = binding energy/Gibbs free energy and N = number of non-hydrogen atoms present in ligand. Among them, CID: 37542 known as Ribavirin (chemical name: 1-D-ribofuranosyl-1H-1,2,4-triazole-3-carboxamide; active ingredient: ribavirin; http://pubchem.ncbi.nlm.nih.gov/compound/ribavirin#section=Top) was the only known drug molecule, which was also mentioned as ‘bioassay active’ in the PubChem Database (AID: 651758). The other six molecules we found to be potential analogs of ribavirin. The GScore for ribavirin was −7.9 kcal/mol, and it formed H-bonds with the majority of the critical residues, such as N100, N128, V144 and R262 (Fig. 2d). To further elucidate the interactions between ribavirin and AphB, we performed molecular dynamics simulations for 20 nanoseconds (ns). The RMSD plot of the backbone atoms displayed a linear deviation after 10 ns, which reveals a stable interaction between ribavirin and AphB (Fig. 3a), while H-bond analysis showed multiple H-bonding interactions between them throughout the simulation period (Fig. 3b). This indicates that the protein-ligand interaction between AphB and ribavirin is stable, the dynamic interaction is presented as a Supplementary movie file.

### Ribavirin binds to wild type AphB but not to its constitutively active form

#### Saturation transfer difference-nuclear magnetic resonance (STD-NMR)

**Figure 4:**
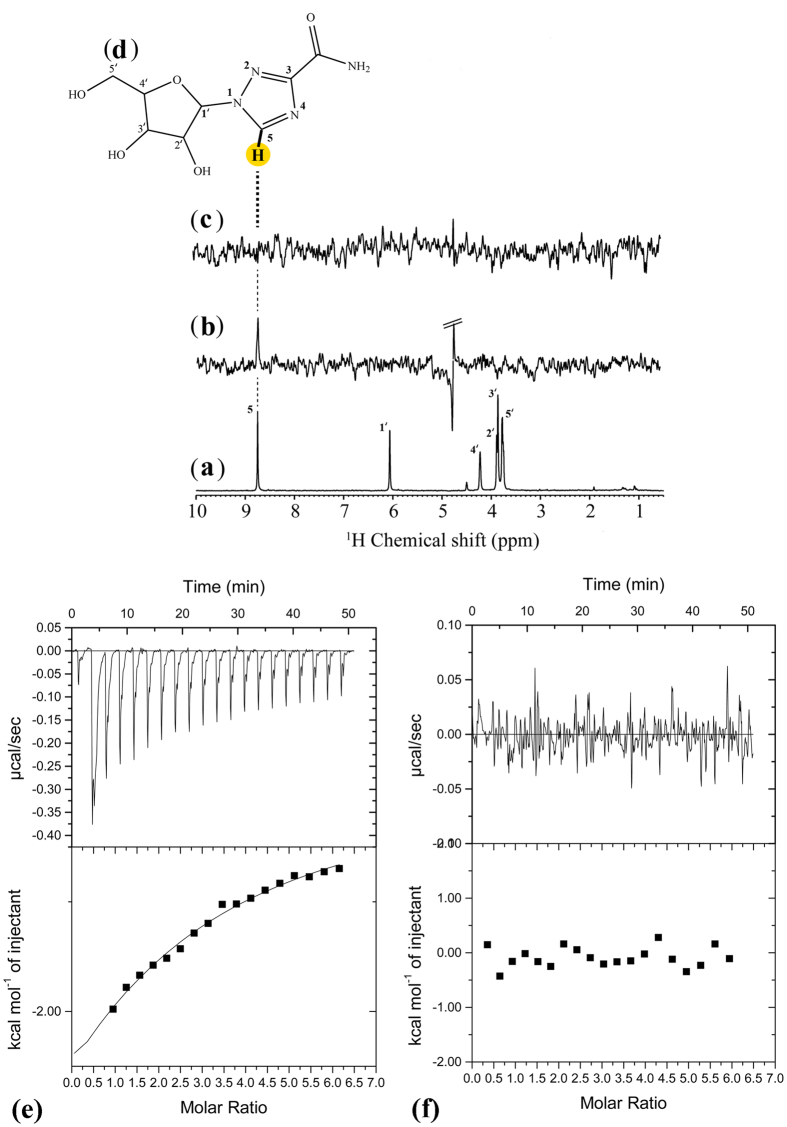
Analysis of AphB binding to Ribavirin by STD-NMR spectroscopy and Isothermal titration calorimetry. NMR experiments were performed on a Bruker AVANCE III 500 MHz NMR spectrometer at 298 K. (**a**) One-dimensional ^1^H NMR Reference spectra of ribavirin. STD spectra of Ribavirin in presence of (**b**) AphB_WT_ protein and (**c**) AphB_N100E_ protein at a molar ratio of protein: ligand = 1:400. (**d**) The STD signal of ribavirin is marked. Isothermal titration calorimetry analysis of the interaction between Aph B_WT_ protein (**e**) and AphB_N100E_ protein (**f**) (50 μM) and Ribavirin (1.5 mM). Heat signals of the ribavirin titration into protein are plotted against against time (top panel) and against the molecular ratio between ribavirin and protein (bottom panel). The best-fit curve corresponds to a single-site binding model.

Previous study had suggested that the wild type AphB, in the absence of effector molecule(s) is unable to activate gene expression; whereas the constitutively active mutant AphB_N100E_ represents a ligand bound conformation of the protein and binds to DNA and activates gene expression^11^. We performed STD-NMR to study the binding interactions of ribavirin with recombinant AphB wild type (rAphB_WT_) and constitutively active mutant (rAphB_N100E_) proteins. This technique identifies the orientation and the mode of binding of chemical groups or residues of ligands that are in close proximity to high molecular weight receptors, where ligands undergo exchange between free and bound states^14^. Complete assignment of ribavirin was performed based on several one-dimensional (1D) ^1^H and ^13^C NMR (DEPT-45, 90 and 135) and 2D homo-nuclear and hetero-nuclear NMR methods (COSY, HET-COR and HSQC) (Data not shown). Figure 4a shows reference NMR spectrum of ribavirin. All the protons of ribose as well as the triazole ring were clearly observed in the spectrum. A strong STD signal was detected at 8.75 ppm for AphB_WT_ protein in the presence ribavirin (Fig. 4b), whereas STD signals were absent for AphB_N100E_ (Fig. 4c). It is noteworthy that the peak at 8.75 ppm corresponds to the hydrogen of the 1,2,4-triazole-3-carboxamide group of ribavirin (Fig. 4d). Since the proton showed close proximity to V144 of AphB in docking studies, STD NMR spectroscopy data complement the docking results (Fig. 2d). The differential STD effects between the wild type and mutant AphB proteins clearly suggest specificity of the above interactions.

#### Isothermal titration calorimetry (ITC)

We performed ITC to study the binding interactions of ribavirin with recombinant AphB proteins, both wild type (AphB_WT_) and constitutively active mutant (AphB_N100E_) (Fig.4e,f). The downward trend in the ITC profile revealed exothermic changes during binding of AphB_WT_ protein (Fig. 4e). The corresponding binding interactions were in the micro-molar (μM) range as evident from the value of equilibrium binding dissociation constant (K_D_ ~ 300 μM). ΔH (−1090 cal.mol^−1^) and ΔS (−20.6 cal.mol^−1^.deg^−1^) that signify the thermodynamic signature of the process suggested that the binding interaction between ribavirin and AphB_WT_ protein was an enthalpydriven (ΔH < 0) and entropically unfavorable (ΔS < 0) process. The negative value of the Gibbs free energy of binding (ΔG ~ −579 cal.mol^−1^) confirmed the spontaneous nature of the interaction. In contrast, the interaction between the mutant protein and ribavirin showed negligible and irregular heat change in ITC thermogram, which proved no binding of the drug to the protein. Overall, the ITC experiment strongly suggests binding between AphB_WT_ and ribavirin as an enthalpy-driven spontaneous process with micromolar binding affinity.

### Ribavirin inhibits cholera toxin production by *Vibrio cholerae* El Tor N16961 strain

**Figure 5:**
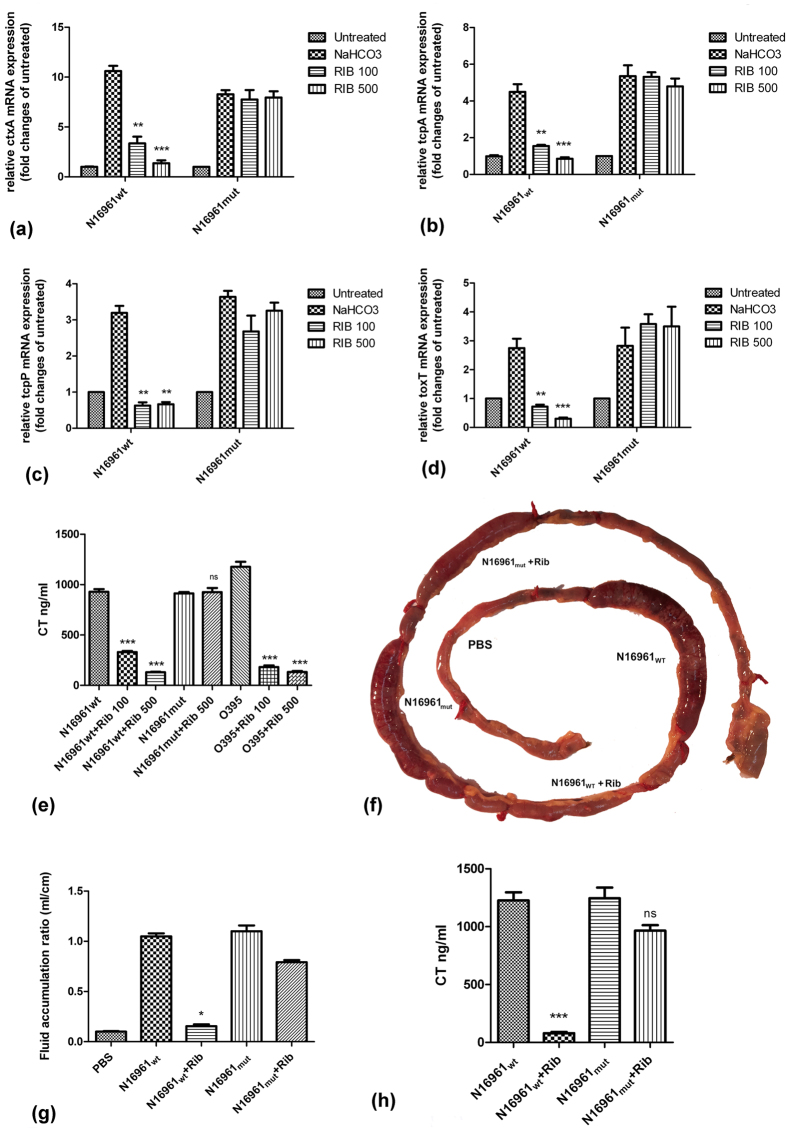
Ribavirin suppresses cholera toxin production by *Vibrio cholerae* both *in vitro* and *in vivo* by targeting AphB. (**a**–**d**) Reverse transcription followed by qPCR showing sodium bicarbonate-stimulated expression of ctxA, tcpA, tcpP, toxT in N16961wt strain normalized against 16 S rRNA. The bacteria were cultured in AKI media for 4 hours in presence or absence of ribavirin (100 μg/ml and 500 μg/ml). Statistical significance was calculated between ribavirin-treated and -untreated among sodium bicarbonate-stimulated samples. (**e**) Cholera toxin concentrations determined by GM1 ELISA in the culture supernatants of N16961 strains and O395 strain. N16961 strains were grown in AKI media for 18 hours in the presence or absence of sodium bicarbonate and ribavirin (100, 500 μg/ml). O395 strain was grown in LB (pH-6.5) overnight at 30 °C. Statistical significance was calculated between ribavirin-treated and -untreated samples. All experiments were done with triplicate for each samples and repeated at least three times. Results of a representative experiment are shown here. Bars refer to mean ± SEM. Statistical significance was calculated by unpaired t-test. (**f**) Rabbit ileal loop assay. Loops were injected with N16961wt or N16961mut strain along with PBS or ribavirin (5 mg). A representative experiment out of three is shown. (**g**) Fluid accumulated in the loops in rabbit ileal loop assay as above. Bars refer to mean ± SEM of three independent animals. Statistical significance was calculated by Kruskal-Wallis test. (**h**) Cholera toxin concentration measured by GM1 ELISA in the fluid accumulated in the rabbit ileal loops under the experiment above. Statistical significance was calculated by unpaired t-test. Statistical significance for e, g, h was calculated between ribavirin-treated and -untreated samples. (*p < 0.05, **p < 0.01, ***p < 0.001 and ns = non-significant).

The above results together suggested that ribavirin bound to the putative ligand-binding pocket of AphB. Given the role of AphB in the regulation of *V. cholerae* virulence genes, we sought to investigate if ribavirin could be a functional antagonist of AphB and inhibit bacterial virulence. To this end, we studied the expression of AphB-regulated virulence genes by log phase cultures of *V. cholerae* El Tor N16961 strain (N16961_wt_). qPCR analysis showed significant suppression of sodium bicarbonate-induced expression of AphB-regulated genes *ctxA, tcpA, tcpP* and *toxT* by ribavirin, suggesting inhibition of AphB functions (Fig. 5a–d). Specificity of ribavirin effects was underscored by its inability to suppress the above genes in a mutant N16961 strain (N16961_mut_) expressing AphB_N100E_, but not expressing AphB_WT_ (Fig. 5a–d). To ensure that ribavirin inhibited the functions of AphB and not its expression, cell lysates were analyzed by western blots, which showed that ribavirin did not alter AphB protein levels (Supplementary Fig. S1a). The above results were further supported by progressive suppression of cholera toxin (CT) production by N16961_wt_ strain, as measured by GM1 ELISA in the bacterial culture supernatants, in the presence of increasing concentrations of ribavirin. The drug failed to inhibit CT production by N16961_mut_ strain, which correlated with its inability to bind to AphB_N100E_ (Fig. 5e). These effects were not specific to a particular *V. cholerae* strain, as ribavirin also inhibited CT production by a classical strain (*V. cholerae* O395) (Fig. 5e). Next, to prove that ribavirin effects could be observed *in vivo*, we checked for CT-induced fluid accumulation in a modified rabbit ileal loop (RIL) assay^15^ after injection of the loop with N16961_WT_ strain (10^8^) along with ribavirin or PBS. Fluid accumulation was markedly reduced by ribavirin (Fig. 5f,g), with significantly diminished concentrations of CT detected in the accumulated fluid (Fig. 5h). That ribavirin exerted its role through the inhibition of AphB was confirmed by replacing N16961_wt_ with N16961_mut_ strain into the loops. In agreement with the *in vitro* experiments, considerable amounts of fluid with CT concentration comparable to the ribavirin untreated loops were accumulated, suggesting significantly less inhibition of AphB_N100E_ functions by ribavirin (Fig. 5f–h). Together these results indicate that ribavirin suppresses *Vibrio cholerae* pathogenesis by inhibiting AphB functions.

### Ribavirin inhibits intestinal colonization of *Vibrio cholerae*

**Figure 6:**
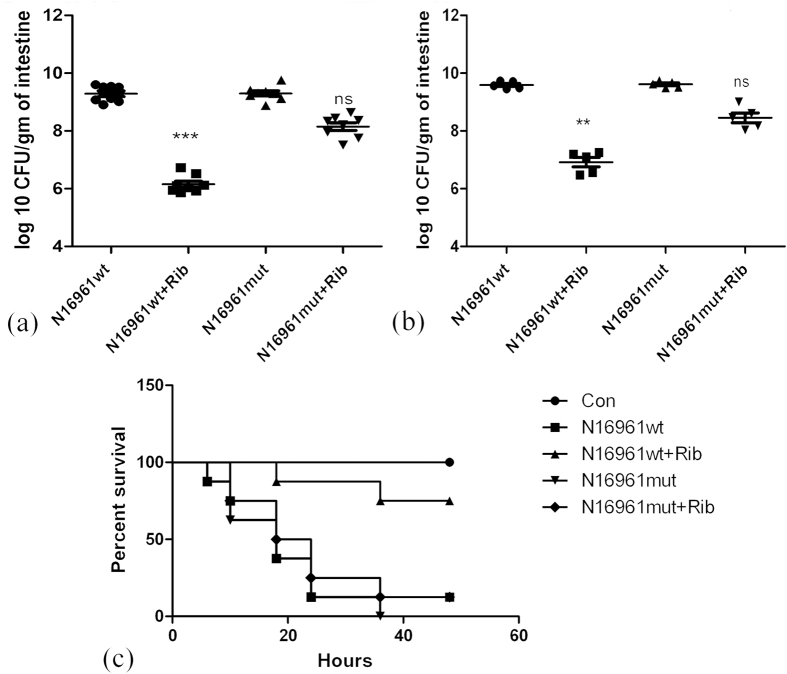
Ribavirin reduces N16961 virulence in suckling mice model: (**a** and **b**) Intestinal colonization of N16961_wt_ and N16961_mut_ strains in 4-5 days old (suckling) Swiss Albino mice infected orally with or without ribavirin treatment (100 mg/kg/mouse). Ribavirin was administered either after 2 hours of infection (**a**) or after 12 hours (**b**). Each dot indicates one mouse. Horizontal bars represent mean CFU counts for all mice used in two independent experiments. Statistical significance was evaluated by Kruskal-Wallis test. Statistical significance for a,b was calculated between ribavirin-treated and -untreated samples. (*p < 0.05, **p < 0.01, ***p < 0.001 and ns = non-significant). (**c**) Survival of suckling mice infected with N16961_wt_ and N16961_mut_ strains, untreated or treated with ribavirin orally (100 mg/kg/mouse). Each group had 8 mice.

Intestinal colonization of *V. cholerae* was studied using a suckling mouse model^16^. We administered bacteria (10^7^ cfu/mouse) into the 4–5 days old Swiss Albino mice through oral gavage, followed after two hours by PBS or ribavirin. Significant decrease in the intestinal colonization of mice by N16961_wt_ strain, but not by N16961_mut_ strain was observed after 18 hours (Fig. 6a). To further reinforce that AphB inhibition by ribavirin was responsible for reduced colonization, we infected the mice with N16961ΔAphB strain and found that it colonized poorly in the mouse intestine. These results are in agreement with previous reports^17^. However, ribavirin treatment displayed no further reduction in the colonization by N16961ΔAphB strain (Supplementary Fig. S2).

To investigate the therapeutic effects of ribavirin administration after colonization of *V. cholerae* in the intestine and the onset of symptoms, suckling mice were treated with it 12 hours after bacterial inoculation. In agreement with our previous results, we found that delayed administration of ribavirin also resulted in decreased recovery of the colonized N16961_wt_ strain, but not the N16961_mut_ strain from the mice intestine (Fig. 6b). Next, we checked if ribavirin could protect suckling mice infected with N16961 strain. For this purpose, the mice were challenged with 5 × 10^7^ cfu of the bacteria followed after 2 hours with oral administration of PBS or ribavirin. Mortality monitored for 48 hours showed that 90% of mice infected with N16961_wt_ strain succumbed, while 75% of them survived if treated with ribavirin. In contrast, ribavirin protected only ~10% of mice infected with the N16961_mut_ strain (Fig. 6c).

### Structural conservation of bacterial LTTRs

Forty three crystal structures of LTTRs with literature reference at NCBI PubMed were available from Protein Data Bank (PDB) at the time of the study (Supplementary Table S1). Out of them, fifteen were unique LTTR proteins present in twelve bacteria (Supplementary Table S2). Further analysis of the proteins revealed their highly diverse primary sequence, while the secondary structures were quite conserved (Supplementary Fig. S3). The tertiary structure of the ligand-binding sites, located at the interface between the RD-I and RD-II domains^11^ are more or less conserved, as revealed from the superimposition of 15 different LTTR proteins (Supplementary Fig. S4). This was further supported by relative RMSD of AphB crystal with other LTTR structures (~2 Å) (Supplementary Table S3) after considering the regions consisting of the active site residues. Although the folding patterns of LTTRs were similar, as evident from superposition of 15 unique LTTR crystal structures, the residues involved in the co-inducer binding domain were different in each case. There were residues which were positional analogs of each other, but the side-chain differences made the binding pocket unique in some cases. To check this, we docked ribavirin with 14 LTTR crystal structures other than AphB and found that ribavirin bound to these LTTRs with different intensities (GScore ranging between −8.26 to −3.86) including some not binding into the co-inducer binding pocket due to the smaller cavity size (Supplementary Fig. S5). We concluded that ribavirin could interact with multiple LTTRs, which had similar active site residues in terms of side-chain orientation and charge density.

### Ribavirin inhibits intracellular survival and systemic infection of *Salmonella* Typhi through the inhibition of LTTR functions

**Figure 7:**
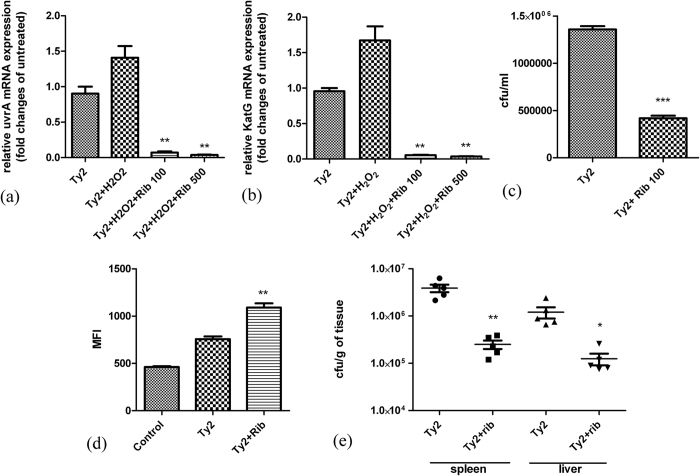
Inhibition of *Salmonella* Typhi virulence both *in vitro* and *in vivo*. (**a** and **b**) Reverse transcription-qPCR analysis showing H_2_O_2_ (600 μM)-induced and 16 S rRNA-normalized expression of *uvrA* and *katG* in S. Typhi Ty2 cultured in LB in the presence or absence of ribavirin (100 μg/ml and 500 μg/ ml) for 4 hours. Statistical significance was calculated between ribavirin-treated and -untreated among H_2_O_2_-stimulated samples. (**c**) CFU counts of intracellular S. Typhi Ty2 strain recovered from THP-1-derived macrophages after 24 hours of culture in the presence or absence of ribavirin. (**d**) ROS production by S. Typhi Ty2 strain infected THP-1-derived macrophages after 18 hours of culture in the presence or absence of ribavirin. ROS was measured using H2DCFDA dye and analyzed by FACS. The mean fluorescence intensity (MFI) is plotted. All experiments were done with triplicate for each samples and repeated at least three times. Results of a representative experiment are shown here. Bars refer to mean ± SEM. Statistical significance was calculated by unpaired t-test. (**e**) Iron-overload BALB/c mice were orally infected with sub-lethal doses (5 × 10^5^) of S. Typhi Ty2 strain with or without oral administration of ribavirin (100 mg/kg/day). CFU counts of bacteria recovered from the spleen and liver after 48 hours of infection were plotted. Horizontal bars represent mean CFU counts for all mice used in one of two independent experiments. Statistical significance was evaluated by Mann-Whitney test. Statistical significance for c, d was calculated between ribavirin-treated and -untreated samples. (*p < 0.05, **p < 0.01 and ***p < 0.001).

Hrg, a *Salmonella* LTTR, is required for protection against oxidative stress and bacterial growth and survival within macrophages. Thus, a mutant *S*. Typhimurium strain deleted of Hrg was found to be more susceptible to H_2_O_2_^18^. To check if ribavirin could bind to Hrg, we performed ITC and found that ribavirin binds to Hrg (K_D_ = 235 μM) (Supplementary Fig. S6). To determine if ribavirin could inhibit the Hrg regulon, *S.* Typhi Ty2 strain were grown in LB containing H_2_O_2_. Expression of the Hrg target genes, *uvrA* and *katG* studied by qPCR was significantly repressed in the presence of ribavirin, whereas Hrg protein levels remained unaltered (Fig. 7a,b, Supplementary Fig. S1b).

Effects of ribavirin on the survival of *Salmonella* Typhi within macrophages were studied in THP1-derived macrophage cells infected with the bacteria. Intracellular CFU counts were significantly less in the ribavirin-treated compared to untreated cells, suggesting inhibition of intracellular survival by ribavirin (Fig. 7c). It was previously reported that Hrg was responsible for quenching of H_2_O_2_ and macrophages infected with Hrg mutant *S*. Typhimurium strain exhibited increased intracellular ROS accumulation^18^. We checked if treatment with ribavirin increased intracellular ROS accumulation in THP1-derived macrophages infected with *Salmonella* Typhi. ROS accumulation as measured by the dye CM-H2DCFDA, which gets converted into its fluorescent form in the presence of ROS was significantly increased in *Salmonella* Typhi-infected cells treated with ribavirin compared with ribavirin-untreated, infected cells (Fig. 7d).

Hrg was reported to be required for the survival of *Salmonella* in the spleen and liver^18^. We investigated the therapeutic role of ribavirin in an iron-overload mouse model of *S.* Typhi infection^19^. Four hours after bacterial infection (10^6^/mouse), PBS or ribavirin (100 mg/kg/day) was administered orally every 12 hours for 2 days. Visceral organs (liver and spleen) collected at the end of the experiment showed significantly reduced bacterial load in the mice treated with ribavirin (Fig. 7e). Together the above findings suggest that ribavirin inhibits the virulence of *Salmonella* Typhi both *in vitro* and *in vivo*.

### Ribavirin suppresses intestinal colonization of EPEC by inhibiting LTTR

**Figure 8:**
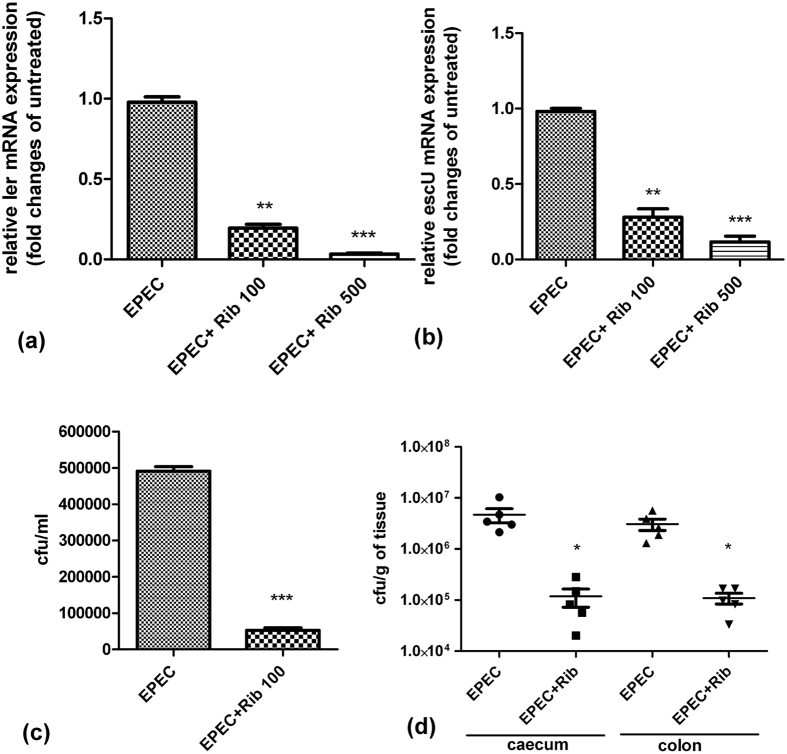
Ribavirin inhibits EPEC virulence both *in vitro* and *in vivo*. (**a** and **b**) Reverse transcription-qPCR analysis showing 16 S rRNA-normalized expression of *ler* and *escU* in EPEC cultured in LB broth in the presence or absence of ribavirin till OD_600_ reached 1. (**c**) EPEC Adherence of EPEC to HT-29 cells in the presence or absence of ribavirin (200 μg/ml) as analyzed by CFU counts. All experiments were done with triplicate samples and repeated at least three times. Results of a representative experiment are shown here. Bars refer to mean ± SEM. Statistical significance was calculated by unpaired t-test. (**d**) CFU counts of EPEC recovered from the caecum and colon of C57BL/6 mice after oral infection followed by ribavirin treatment with. Each dot indicates one mouse. Horizontal bars represent mean CFU counts of all mice from one of two independent experiments. Statistical significance was evaluated by Mann-Whitney test. Statistical significance was calculated between ribavirin-treated and -untreated samples. (*p < 0.05, **p < 0.01 and ***p < 0.001).

Adhesion of EPEC and EHEC to the intestinal epithelial cells is regulated by a chromosomal pathogenicity island termed the locus of enterocyte effacement (LEE), which encodes an adhesin (intimin) and its receptor (Tir) and a T3SS apparatus and its effectors. LTTRs namely QseA, LeuO and LrhA regulates the LEE genes and the flagellar regulon, and is involved in the microcolony formation and adherence to the epithelial cells^20^,^21^,^22^,^23^. To determine if ribavirin could inhibit LEE genes expression, expression of the target genes *ler*, a master regulator of LEE and *escU* in the *in vitro* cultures of EPEC was quantified by qPCR. Gene expressions were significantly repressed by ribavirin (Fig. 8a,b).

To investigate the role of ribavirin on epithelial adhesion of EPEC, which promotes microcolony formation, we cultured HT-29 cells infected with EPEC in the presence of ribavirin. After removal of the non-adherent organisms, the numbers of bacteria attached to the cells were quantified by plating the cell lysates. We observed significant reduction in epithelial adherence of EPEC in the presence of ribavirin (Fig. 8c).

Next, we investigated if ribavirin was capable of inhibiting colonization of the mouse intestine by EPEC. Seven to eight weeks old, male C57BL/6 mice were orally inoculated with bacteria (10^9^ cfu/mouse), followed after 4 hours by oral administration of PBS or ribavirin, repeated every 12 hours. On day 3, mice were sacrificed and colonization of the caecum and colon was studied by CFU counts. Oral administration of ribavirin resulted in nearly 2-log decrease in the intestinal colonization of EPEC (Fig. 8d). However, we did not find any considerable binding of ribavirin to QseA in ITC experiment, suggesting other LTTR targets for the drug (data not shown).

### Effect of Ribavirin on bacterial growth

Ribavirin is a broad-spectrum anti-viral drug that inhibits viral replication^24^. To check the effects of ribavirin on the growth of *Vibrio cholerae* El tor strain N16961, *Salmonella* Typhi Ty2 and EPEC, bacteria were cultured in the respective medium containing various concentrations of ribavirin. Bacterial growth was monitored over time by CFU counts. No significant changes in the log phase growth of any of the bacterial strains were observed in the presence of ribavirin (Supplementary Fig. S7a–c). Taken together the above results indicate that the effects of ribavirin on *V. cholerae, S.* Typhi and EPEC infections are mediated through the inhibition of LTTR functions.

## Discussion

Bacterial pathogens often develop resistance to antibiotic drugs, which target their growth or viability. In contrast, strategies that specifically target virulence pathways which are non-essential for growth would limit selection of the resistant strains, and are candidates for the development of next-generation antimicrobial therapeutics^6^,^25^. Based on this principle, many bacterial transcriptional regulators have been targeted for inhibitor designing^26^,^27^,^28^,^29^,^30^. A small molecule compound virstatin inhibits dimerization of ToxT protein required for transcriptional regulation and expression of cholera toxin and TcpA of *Vibrio cholerae*^26^,^29^. Bacterial LTTRs are ideal targets for designing anti-virulence drugs due to their role in global transcriptional regulation of virulence genes. This would minimize drug resistance due to the structural conservation of the ligand-binding pocket, multiplicity in a single bacteria and their role in unrelated virulence mechanisms^7^. Ligand/co-inducer binding to the C-terminal domain of LTTR facilitates RNA polymerase recruitment to initiate transcription. This implies that a compound, which competes with the ligand for binding to the active site may inhibit LTTR-mediated transactivation. Thus, antagonists were designed based on the structure and binding properties of known ligands of LTTRs. However, *Pseudomonas aeruginosa* PqsR (MvfR) is the only LTTR successfully targeted for inhibitor designing till date^31^. Researchers generated analogs of PqsR ligands to antagonize the protein^32^,^33^,^34^. However, the inhibitor molecules developed by diverse approaches need to successfully clear the tests for bioavailability, pharmacokinetic and pharmacodynamic stability, toxicities etc before being considered as potential drug candidates and put to clinical trials. Large funding support is necessary and several years would elapse before candidate drugs may be screened to get one successful hit. In the present study, we took a different approach, called fragment-based *in silico* designing of small-molecule inhibitors for AphB, a *Vibrio cholerae* LTTR and a master regulator of the virulence genes, since the ligand/co-inducer of AphB is not known and currently, there is no knowledge regarding the molecular structure that could accommodate the active site of AphB. This is an established approach for structure-guided drug designing where no information about the ligand is available and delivers molecules with high specificity and selectivity and reduces off-target binding, leading to less untoward side effects of the designed drugs^35^. We filtered the fragments at the beginning and at each subsequent step based on their interactions with two critical active site residues of AphB, N100 and R262. Such interactions would inhibit transactivation by AphB by preventing bending of the molecule that would allow the residues to come closer^11^. Our approach to start with FDA-approved drug fragments had distinct advantages, since the subsequent step of fragment joining to design interactors of the AphB ligand-binding pocket would be capable of identifying commercially available drug molecules or at the least, synthesizable compounds and their structural analogues with comparable or better binding efficacy and higher bioactivity. Substructure-based searches for already available compounds with structural and functional similarities to the linked-fragments led us to identify ribavirin, which binds to the co-inducer binding pocket of AphB with high ligand efficiencies. This is an example of ‘drug repurposing’ that may explore novel targets for the existing drugs or their modified versions and greatly reduce the duration and cost of drug development. In a recently-published review, eleven existing drugs were listed, which are currently used for other diseases, but possess additional antibacterial activities^36^. However, a more straight-forward approach of virtual screening of the drug library identified several other molecules with comparable or higher GScores than ribavirin (DB00811) (supplementary Table S4). Since a high GScore may be accompanied by a poor ligand efficiency that precludes efficient binding, we would have to physically screen all the drug molecules with GScores above an arbitrary threshold level if we followed the virtual screening approach. A potential limitation of our substructure-based screening strategy though is the inability to predict completely novel compounds, which require a chemical synthesis approach.

Ribavirin, a synthetic triazole compound and a guanosine analogue, inhibits replication of a wide range of DNA and RNA viruses *in vitro*. However, the effective inhibitory concentration varies considerably, depending on the virus tested and the cell lines used. The IC_50_ dose of ribavirin for viral replication ranges between 20 and 200 μM in most instances^37^, but the anti-HCV activity may require a higher dose (100 μg/ml)^38^. At equivalent doses, ribavirin inhibited LTTRs in bacterial cultures and intracellular survival of *S*. Typhi within cultured macrophages. We and others found no cytotoxicity at this or even much higher concentrations of ribavirin^37^,^38^. Finally, ribavirin did not display any direct bacteriostatic or bactericidal properties at concentrations as high as 5 mg/ml.

*In vivo* efficacy of ribavirin was reported against several RNA viruses, although the drug is currently recommended only against severe RSV infection^39^, and in combination with interferon-α for patients suffering from chronic HCV infection^40^. In animal models, ribavirin (50–100 mg/kg/d) significantly inhibited viral replication and improved survival^41^,^42^,^43^,^44^. At similar doses, ribavirin displayed LTTR inhibitory effects *in vivo* and suppressed bacterial pathogenesis.

As opposed to injectable ribavirin used in most animal studies^41^,^43^,^44^, we administered it orally that is widely preferred in clinical settings. The LD_50_ oral dose of ribavirin in mice is twice (2 g/kg) as that of the intraperitoneal dose (0.9–1.3 g/kg) (https://pubchem.ncbi.nlm.nih.gov/compound/ribavirin#section=Top). Intriguingly, *in silico* binding affinity of ribavirin and its analog ICN17261, which demonstrated significantly reduced toxicity in rats^45^ to AphB was comparable (data not shown).

Despite considerable sequence diversity over the C-terminal domains of LTTRs, three-dimensional structural superimposition suggests that LTTRs have a conserved ligand-binding pocket. It was earlier reported that the residues S99 and A101 of TsaR and V97 and S99 of BenM, which participate in direct interactions with their respective ligands are at analogous positions to P98 and N100 of AphB^46^,^47^. Due to the diversity in sequence, these proteins have different residues at their active site, leading to diverse specificities towards co-inducer molecules or ligands. cis,cis-muconate and benzoate are the two inducer molecules that bind to the primary and secondary effector-binding sites of BenM and activate transcription. Despite having 59% sequence identity with BenM, CatM, another LTTR also binds to cis,cis-muconate to activate transcription. However, it is unable to bind to benzoate due to different active site residues^48^. Thus, the ligands may differ for different LTTRs, but some ligands may have multiple LTTR targets, which have similar folding patterns.

Our computational analysis predicted that ribavirin strongly binds to AphB (GScore −7.9 kcal/mol) and forms H-bonds with N100, N128, A130, D131, V144 and R262 residues of the ligand-binding pocket. This prediction was validated by NMR studies, which suggested ribavirin-binding to AphB, but not to its constitutively active mutant (AphB_N100E_). However, NMR showed a distinct peak at 8.75 ppm corresponding to the hydrogen of the 1,2,4-triazole-3-carboxamide group, but not to the other protons attached to the −OH or −NH_2_ group of the drug molecule. This may be explained by the fact that the experiment was conducted in D_2_O only due to the buffer/water solubility of AphB. Because of the labile nature, NMR signals corresponding to the 2′-, 3′- and 5′-hydroxyl or amide protons of ribavirin were absent in proton NMR and this phenomenon may be attributed to deuterium exchange. Although in ITC experiments, the K_D_ value of the interaction was in the higher micromolar range (~300 μM), suggesting modest affinity between the molecules, specificity of the interaction is underscored by the absence of interaction between AphB_N100E_ and ribavirin. Much higher K_D_ values (2480 μM to undetectable) were reported for the interactions between viral RNA-dependent RNA polymerases and ribavirin, although some of these enzymes are definitive targets of the drug^49^,^50^. It is evident from these studies that ribavirin inhibits the function of viral RNA polymerases despite having a low binding affinity for them.

During infection of infant mice with El Tor biotype, mutations in TcpPH or ToxR failed to abolish *tcpA* and *ctxAB* expression, and hence colonization and pathogenesis. However, mutations of both TcpPH and ToxR completely abolished *tcpA* and *ctxAB* expression^51^. Independent *in vitro* studies had shown that AphB regulates TcpPH and ToxR expression and TcpPH along with ToxRS regulates *ctxAB* and *tcpA* production *in vitro*^9^,^52^. As a result, inhibition of AphB by ribavirin or its deletion suppressed colonization of *V. cholerae* ElTor and fluid accumulation. This is supported by marked transcriptional inhibition of AphB target genes, *ctxAB* and *tcpA* in El Tor biotype strains. In accordance with the binding experiments, functions of only AphB_WT_, but not AphB_N100E_ was inhibited by ribavirin. This provided further support to AphB-ribavirin interaction at the ligand-binding pocket and its functional relevance. Together the above results strongly indicate that ribavirin may exert its role by competing with the putative ligand for AphB binding.

Distribution of LTTRs across bacterial species and structural homology between them suggest wider role for ribavirin in bacterial infections. This was experimentally validated in our study by the inhibition of *S.* typhi and EPEC pathogenesis, perhaps by suppressing LTTRs. Though we excluded the off-targets of ribavirin in *Vibrio cholerae* but we could not do the same in *S.* typhi and EPEC, due to the inability to make structural mutants of LTTRs owing to the absence of crystal structures.

EPEC is a major cause of watery diarrhoea in the neonates and young children worldwide. LTTR family proteins, such as QseA^21^, LeuO^23^ and LrhA^22^ regulate the locus for enterocyte effacement (LEE), which is critical for intestinal colonization and pathogenesis of EPEC and EHEC. Although we failed to identify the LTTR protein which was inhibited by ribavirin, we found that the drug inhibits the expression of LEE genes and colonization of mouse small intestine by EPEC. Since LEE is also required for the pathogenesis of other bacteria like Enterohemorrhagic *E. coli*^20^, ribavirin may have a wider therapeutic application against *E. coli* infections.

Ribavirin also showed efficacy against intracellular pathogens, such as *Salmonella* Typhi. Phagosomal survival of the bacteria within cultured macrophages was compromised by ribavirin treatment, perhaps due to the inhibition of Hrg, an LTTR that confers protection against oxidative stress^18^. A lethal systemic infection of mice with S. Typhi mimicking typhoid fever in humans^19^ was also attenuated by ribavirin treatment. It would be interesting to investigate if ribavirin could be effective against infection by the rapidly-spreading multidrug resistant H58 strain of *S*. Typhi^53^. However, we did not exclude other possible LTTR targets or other functions, such as immunomodulatory role^54^ of ribavirin that might contribute to its anti-infective role against EPEC and *S*. Typhi.

In conclusion, the present study employed bioinformatic and experimental approaches to explore a novel role for ribavirin against bacterial infections through the inhibition of LTTRs. Because of their wide distribution in pathogenic bacteria and diverse role in virulence, LTTRs may be highly effective targets for anti-infective therapy. In addition, this approach is able to design efficient, highly specific and potentially synthesizable ligands for novel therapeutic targets. The newly available molecules would have predictable bioavailability, stability, pharmacokinetic and pharmacodynamic properties and toxicities because of their derivation from already existing drugs. This would greatly shorten the time and minimize costs for the development of new drugs.

## Materials and Methods

### Ethics Statement

All animal experiments were conducted following the protocols approved by the Institutional Animal Ethics Committee of National Institute of Cholera and Enteric Diseases (NICED) registered under ‘Committee for the Purpose of Control and Supervision of Experiments on Animals’ (CPCSEA), Government of India (Registration No. 68/1999/CPCSEA dated 11-03-1999).

### Structural analysis & inhibitor designing

LTTR crystal structures were downloaded from Protein Data Bank (PDB). Secondary structural alignment was performed using DALI server (http://ekhidna.biocenter.helsinki.fi/dali_server) and the 3D structural alignment was performed in Discovery Studio (DS 2.5) software. AphB from *Vibrio cholerae* (PDB ID: 3SZP) was selected for inhibitor designing. 1491 commercial fragments extracted from the FDA approved drugs as on 04-02-2014 was downloaded from e-LEA3D: Chemoinformatic Tools and Databases (http://chemoinfo.ipmc.cnrs.fr/lea.html). Fragment-based ligand designing was done in DS 2.5 using LUDI, an empirical scoring function based method^55^, using default parameters^12^. At each step, selection of fragments was done based on critical interactions with the active site residues of AphB and the free energy of binding. This was followed by searches for molecular substructures of the designed ligands in the PubChem database to generate a small-molecule library of similar compounds. Only the drug-like compounds were considered further for molecular docking to the AphB co-inducer- binding site using GlideXP method and hits were prioritized based on respective Ligand Efficiency (LE) calculation. To further validate the interaction stability, we performed 20 ns molecular dynamic (MD) simulation of the AphB-ligand complex in solution using GROMACS4.5 software^56^. The parameters for the simulation were set as per our previous publication^57^. The root mean square deviation (RMSD) and the H-bond interaction plots were generated from the trajectory output file using Microsoft Excel program. Molecular interactions were visualized through Discovery Studio software.

### Cells, bacterial strains and reagents

Cell lines were procured from American Type Culture Collection (ATCC). Cell culture reagents were purchased from Invitrogen and bacterial culture media from BD Difco. Ribavirin was purchased from Sigma-Aldrich. Restriction enzymes were purchased from New England Biolabs. Bacterial strains *Vibrio cholerae* El tor strains N16961, Enteropathogenic *E.coli, E.coli* DH5α-λpir, *E.coli* SM10λ-pir were provided by Dr.T. Ramamurthy (NICED, India). S.Typhi Ty2 was gifted by J. Parkhill (Sanger Institute, Hinxton, United Kingdom). Plasmid pBAD24 was gifted by Dr. A.K. Mukhopadhyay (NICED, India). Primers (Supplementary Table S5) were custom synthesized from IDT.

### Cloning, expression and purification of AphB and N100E AphB

ORF of AphB was PCR-amplified from genomic DNA of N16961 strain, ORF of Hrg was amplified from Ty2 strain and ORF of QseA was amplified from EPEC strain, cloned into pTZ57R/T and sub-cloned into pET28a (Novagen) and pBAD24 expression vectors. Constitutively active AphB mutant (N100E) was generated by site-directed mutagenesis (Agilent technologies) as per the manufacturer’s protocol and confirmed by sequencing. Expression plasmids of AphB_WT_, AphB_N100E_, Hrg and QseA were transformed into *E.coli* strain BL21 (DE3) (Novagen). BL21(DE3) was cultured at 37 °C till the OD_600_ reached 0.6. Protein expression was induced with 0.1 M IPTG at 37 °C for 4 hours and purified using Ni-NTA agarose columns (Qiagen). The recombinant proteins were dialyzed against 10 mM sodium phosphate buffer containing 50 mM NaCl (pH-7.4).

### STD NMR

STD NMR experiments were performed on a Bruker AVANCE III 500 MHz NMR spectrometer, equipped with a 5 mm SMART probe at 298 K. Data acquisition and processing were carried out using Topspin^TM^*v3.1* software suite^58^. For all NMR experiments, deuterated buffer was used. Proteins were used at a concentration of 5 μM and ribavirin at 2 mM. For 1D STD experiment, on-resonance irradiation frequency of the protein (AphB) was set at 7.2 ppm, where only the protein resonance, but no ligand resonance was present. Similarly, off-resonance irradiation was set to 40 ppm where no protein or ligand signals were detected. The standard STD pulse sequences with WATERGATE 3-9-19 sequence for water suppression was used. Selective saturation of protein was achieved using a cascade of 40 Gaussian- soft pulses (49 ms each) separated by an inter-pulse delay of 1 ms for a total saturation time of 2 s. Internal subtraction of the two spectra (on-resonance and off resonance) by phase cycling leads to the difference spectrum that contains ligand signals attenuated via saturation transfer from protein. Total number of scans was 512, and 16 dummy scans using 12 ppm spectral widths was done for the 1D STD NMR spectra.

### Isothermal titration calorimetry

ITC experiments were performed on VP-ITC micro-calorimeter (GE health care) at 25 °C. Ribavirin was dissolved in the same buffer as the recombinant proteins^59^. All samples were degassed before experiment. The titration was performed by using 50 uM protein in ITC cell and 1.5 mM ribavirin in a syringe with 250 rpm stirring speed, and an initial delay of 60 sec. The reference power was fixed at 10 μcal/sec to retain a flat base-line during titration. A total of 20 injections were made with the initial one of 0.4 ul and the rest of 2.0 ul each with a spacing of 150 sec and 5 sec filter period. The control experiment was performed using the buffer solution into ITC cell to subtract the heat of dilution of the drug in buffer keeping the same experimental parameters. The raw data were plotted using Micro Cal Origin 7.0 software and single site binding model was used to calculate binding constants. Other relevant thermodynamic parameters were calculated using fundamental equations of thermodynamics as mentioned earlier.

### Generation of N16961 mutant strains expressing AphB_WT_ and AphB_N100E_

AphB deletion mutant of N16961 strain was generated using the suicide vector pCVD442 (Addgene). Gene fragments flanking upstream and downstream of AphB were amplified, ligated into pCVD442 and transformed into SM10λ-pir. The transformed SM10λ-pir was conjugated with N16961 and the conjugates were selected in TCBS agar containing ampicillin. Gene deletion was confirmed by PCR. Deletion mutants of N16961 (N16961ΔAphB) were electroporated with AphB_WT_ or AphB_N100E_ cloned in pBAD24.

### Suckling mouse assay

4–5 days old Swiss albino mice were inoculated orally with 10^7^ cfu of bacterial cells resuspended in 50 μl of PBS^16^. Two hours or 12 hours later, 20 μl of PBS or ribavirin (100 mg/kg/mouse) was administered orally. Mice were maintained at 30 °C and sacrificed after 16 hours. The entire intestine was removed, weighed, homogenized in PBS and was serially diluted and plated on LB agar containing streptomycin.

### Rabbit ileal loop assay

The RIL assay was performed in New Zealand white rabbits (2 kg) as previously described^15^. Loops were inoculated with bacteria (10^8^ CFU) resuspended in PBS with or without ribavirin (5 mg). Rabbits were sacrificed after 18 hours and fluid accumulation per unit length of the loops was measured.

### Intracellular survival assay

Human acute monocyte leukemia cell line THP-1 was maintained in RPMI 1640 containing 10% FBS. Cells were differentiated and infected as previously described^60^. Post infection medium was replaced with complete RPMI1640 containing gentamicin (10 μg/ml) and ribavirin (100 μg/ml). Cells were lysed after 24 hours with PBS containing 0.25% Triton X-100 and plated on LB agar and the intracellular CFU was counted.

### Infection of mice with *Salmonella* Typhi

An iron-overload mouse model was used for oral *S*. Typhi infection^19^. Eight to ten weeks old BALB/c mice were intra-peritoneally injected with Fe^3+^ (0.32 mg/g of mice) and Desferrioxamine (Novartis) (0.025 mg/g of mice) 4 hrs prior to the bacterial challenge. Mice were orally inoculated with *S*. Typhi. Four hours later, ribavirin (100 mg/kg/mouse) was administered orally every 12 hours. 48 hours after infection, spleen and liver were collected, weighed and homogenized in PBS containing 0.5% Triton-X 100 and serial dilutions were plated on Hektoen enteric agar plates.

### EPEC adherence to HT-29 cells

HT-29 cells were maintained in DMEM supplemented with 10% FBS. Cell monolayers were grown in 24-well tissue culture plates to obtain a 60% confluence. Cells were infected with bacterial suspensions in DMEM at a MOI of 100 for 3 hours in the absence of antibiotic, in presence or absence of ribavirin (100 μg/ml). Cells were washed thrice with PBS to remove the non-adherent bacteria, lysed with 0.25% triton-X 100 for 30 minutes and the lysates were diluted and plated on MacConkey agar containing ampicillin.

### Infection of mice with EPEC and *in vivo* adherence assay

7–8 weeks old C57Bl/6 male mice were used for the study^61^. Mice were given water containing 5 g/L streptomycin for 24 hours followed by water without the antibiotic for 24 hours. Mice were starved overnight and next morning, EPEC suspended in PBS were administered orally. Four hours after the challenge, food and water were resumed and ribavirin was given orally (100 mg/kg/day), every 12 hours. On day 3, mice were sacrificed to collect the caecum and colon. Colonization was studied by plating the homogenized tissue on McConkey agar containing ampicillin.

### Gene expression analysis

*Vibrio cholerae N16961* was cultured in AKI media supplemented with sodium bicarbonate, while *Salmonella* Typhi was grown in Luria Bertani (LB) broth in presence of H_2_O_2_ and EPEC in LB alone. Cultures were grown in the presence and absence of ribavirin. Total RNA was extracted using the TRIzol reagent (Invitrogen) according to the manufacturer’s protocol and was treated with DNase I (NEB). DNA-free RNA was used for cDNA synthesis using SuperScript II reverse transcriptase. Quantitative PCR (qPCR) was performed in a StepOnePlus system (ABI) using SYBR green master mix. Relative quantitation was done by the comparative threshold cycle (*CT*) method. The levels of expression of the genes of interest were normalized against 16 S rRNA using the formula 2^−ΔΔCT ^62. The protein levels of AphB and Hrg in the cultures were analyzed by Western blot of cell lysates using anti-serum directed against AphB and Hrg.

### GM1 ELISA

The amount of CT in the bacterial cell supernant and fluid accumulated in rabbit ileal loop was determined by ELISA. Briefly ELISA plates were coated with 100 ng/well of GM1 ganglioside (Sigma). 100 μl of cell supernatant or intestinal fluid was used. As primary antibody rabbit polyclonal anti-CT antibody (Sigma) was used. Anti-rabbit IgG conjugated with HRP (Pierce) was used as secondary antibody. Purified CT (Sigma) was used to plot the standard curve.

### Bacterial growth curve measurement in presence of Ribavirin

10 ml of culture media supplemented with or without ribavirin was inoculated with 100 μl bacterial cultures (OD600 = 1.0) and incubated at 37 °C under shaking conditions. Bacterial growth was monitored by sampling every 2 hrs followed by CFU counts after overnight growth on LB agar containing antibiotics.

## Additional Information

**How to cite this article**: Mandal, R. S. *et al*. Ribavirin suppresses bacterial virulence by targeting LysR-type transcriptional regulators. *Sci. Rep.* **6**, 39454; doi: 10.1038/srep39454 (2016).

**Publisher's note:** Springer Nature remains neutral with regard to jurisdictional claims in published maps and institutional affiliations.

## Supplementary Material

Supplementary Information

Supplementary Movie
