# Supplementary material for: Ribavirin suppresses bacterial virulence by targeting LysR-type transcriptional regulators
Source: Sci Rep. 2016 Dec 19;6:39454. doi: 10.1038/srep39454 (PMC5171790; doi:10.1038/srep39454)
Supplement: Supplementary Information [file srep39454-s1.pdf]

Ribavirin suppresses bacterial virulence by targeting LysR-type transcriptional regulators

Rahul Shubhra Mandal[1#], Atri Ta[2#], Ritam Sinha[3], Nagaraja Theeya[2], Anirban Ghosh[4], Mohsina Tasneem[1], Anirban Bhunia[4], Hemanta Koley[3], Santasabuj Das[1,2*]

Supplementary movie

Dynamic interaction of ribavirin within AphB co-inducer/ligand binding site, as evident from 20ns molecular dynamic simulation.

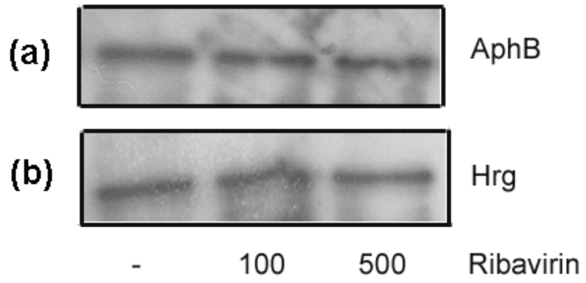

Supplementary figure S1. Ribavirin does not alter the expression of AphB of N16961$_{wt}$ (a) and Hrg of S. Typhi Ty2 strains *in vitro* as shown by western blot: Bacterial strains, N16961 (a) and S. Typhi Ty2 (c) were cultured in AKI media or LB at 37°C with shaking in the presence of increasing concentrations of ribavirin.

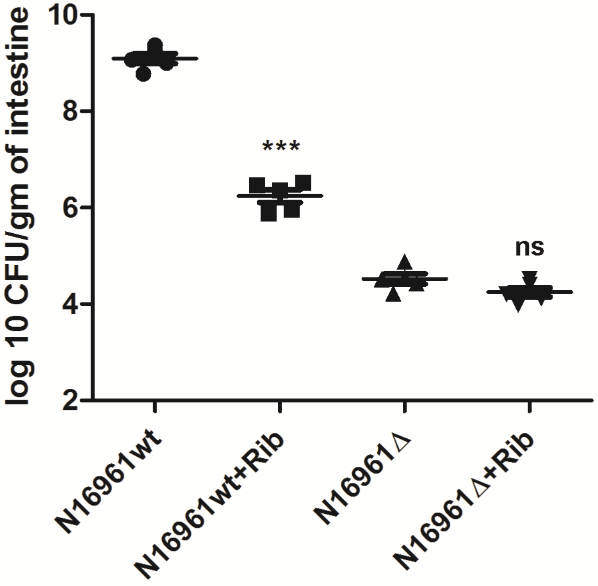

Supplementary figure S2. Ribavirin does not affect N16961ΔAphB strain virulence in suckling mice model**:** Intestinal colonization of $N16961_{wt}$ and N16961ΔAphB strains in 4-5 days old (suckling) Swiss Albino mice infected orally with or without ribavirin treatment (100 mg/kg/mouse). Ribavirin was administered either after 2 hours of infection. Each dot indicates one mouse. Horizontal bars represent mean CFU counts for all mice used in two independent experiments. Statistical significance was evaluated by Kruskal-Wallis test. Statistical significance for a,b was calculated between ribavirin-treated and -untreated samples. (*p<0.05, **p<0.01, ***p<0.001 and ns=non-significant).

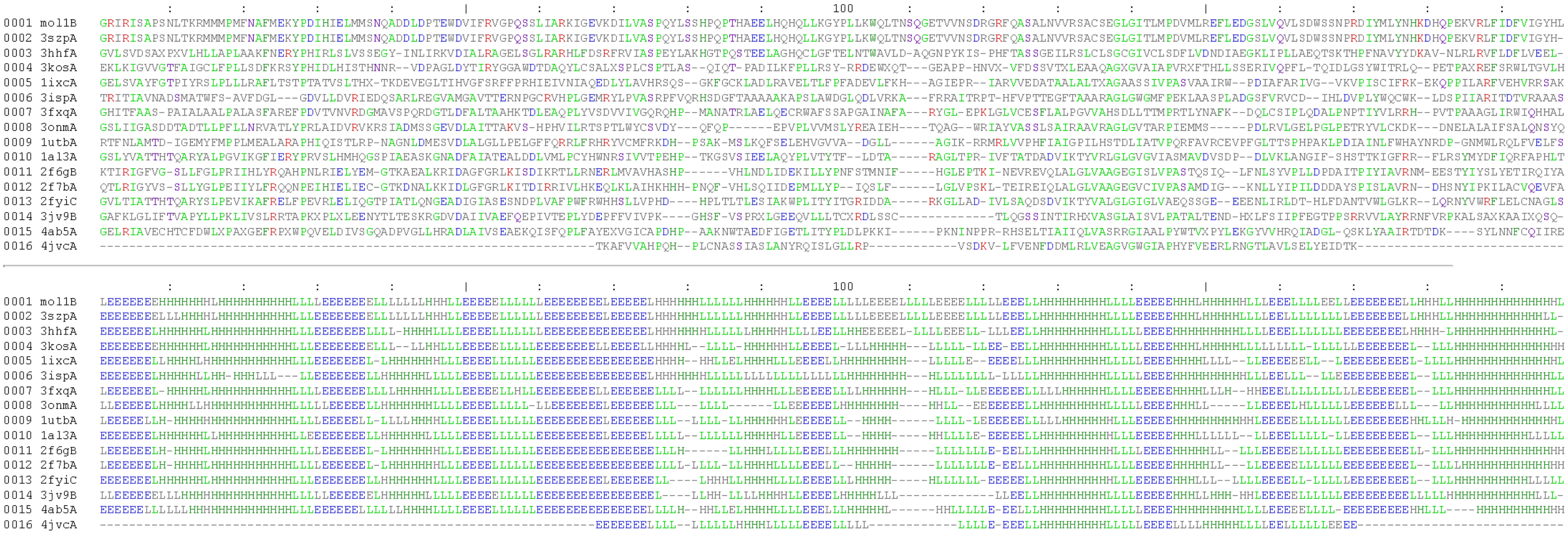

Supplementary figure S3. (a) Sequence alignment of the N-terminal 145 residues of LTTR proteins. (b) Secondary structure alignment of the LTTR proteins, where E denotes β sheet, H denotes α helix and L denotes loop.

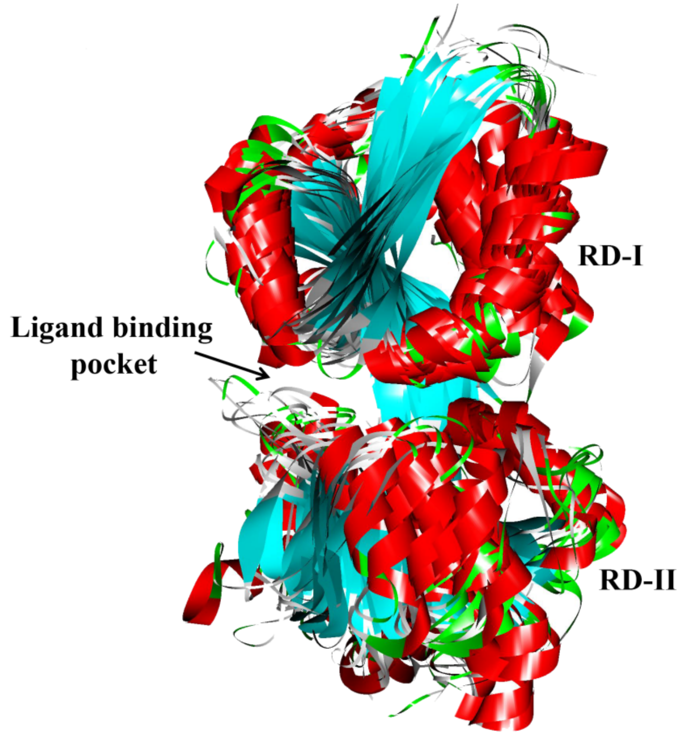

Supplementary figure S4. Superimposition of the truncated N-terminal 145 residues from the crystal structures of fifteen LTTR proteins as listed in supplementary table S2, showing a conserved ligand/co-inducer-binding pocket.

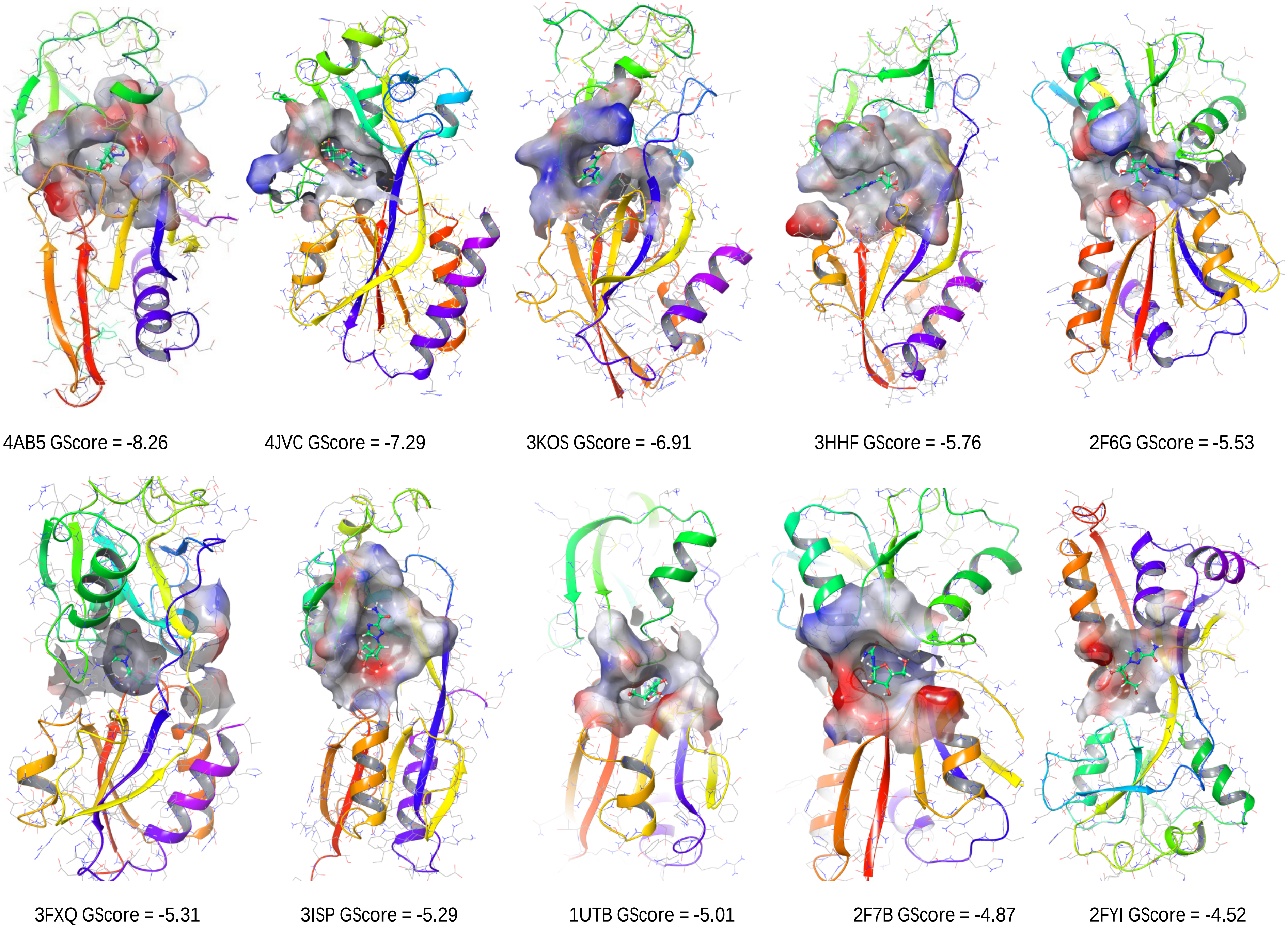
4AB5 GScore = -8.26
4JVC GScore = -7.29
3KOS GScore = -6.91
3HHF GScore = -5.76
2F6G GScore = -5.53
3FXQ GScore = -5.31
3ISP GScore = -5.29
1UTB GScore = -5.01
2F7B GScore = -4.87
2FYI GScore = -4.52

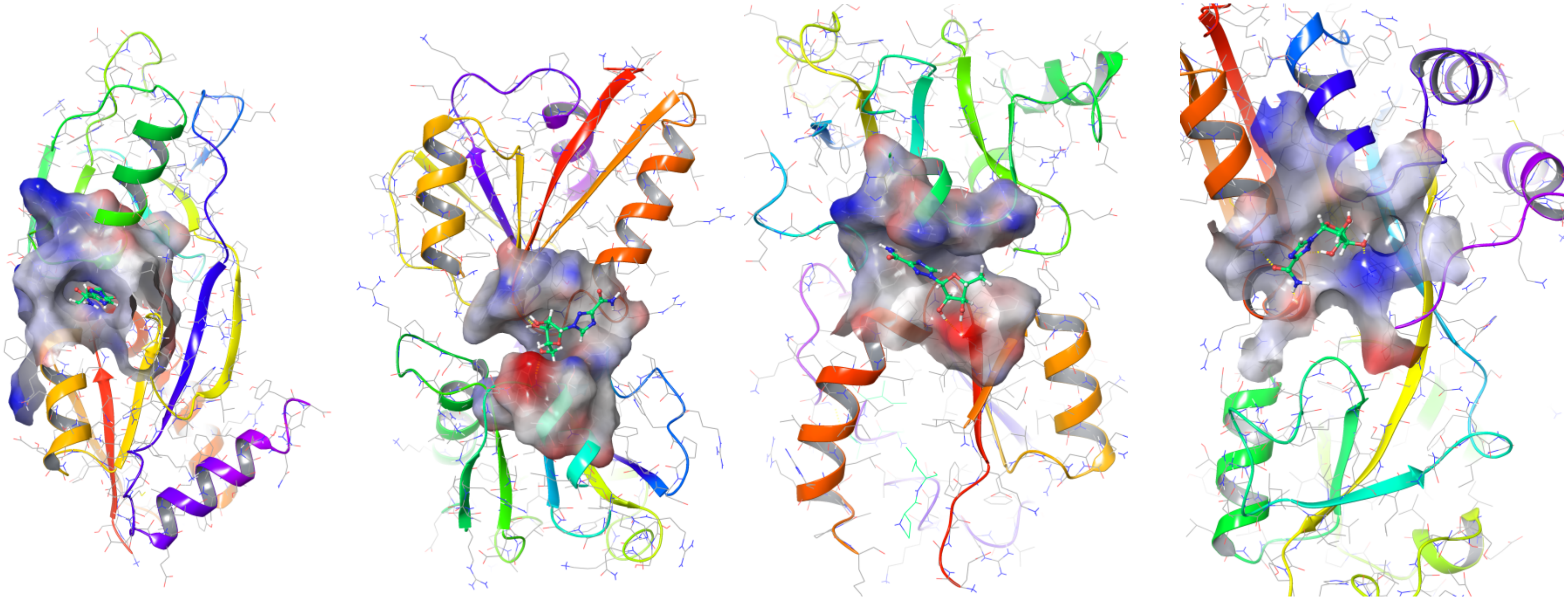

3ONM GScore = -4.51 | 1IXC GScore = -4.35 | 3JV9 GScore = -3.93 | 1AL3 GScore = -3.86

Supplementary figure S5. Docking of ribavirin with 14 LTTR crystal structures showing the corresponding binding pose within the AphB co-inducer binding site. The PDB id for each of the crystal structure and the respective Glide GScores are mentioned ate the bottom of each image.

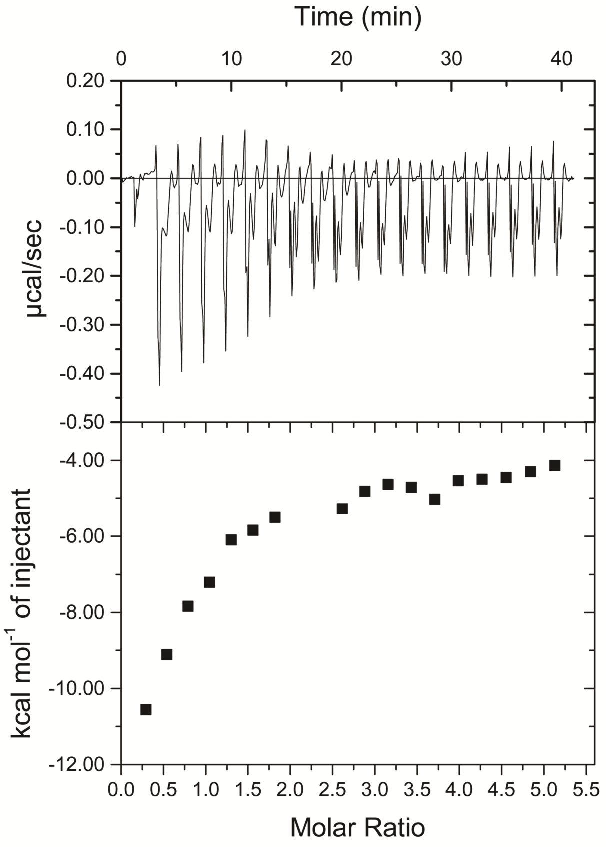

Supplementary figure S6. Isothermal titration calorimetry analysis of the interaction between Hrg (50 µM) and Ribavirin (1.5 mM). Heat signals of the ribavirin titration into protein are plotted against against time (top panel) and against the molecular ratio between ribavirin and protein (bottom panel). The best-fit curve corresponds to a single-site binding model.

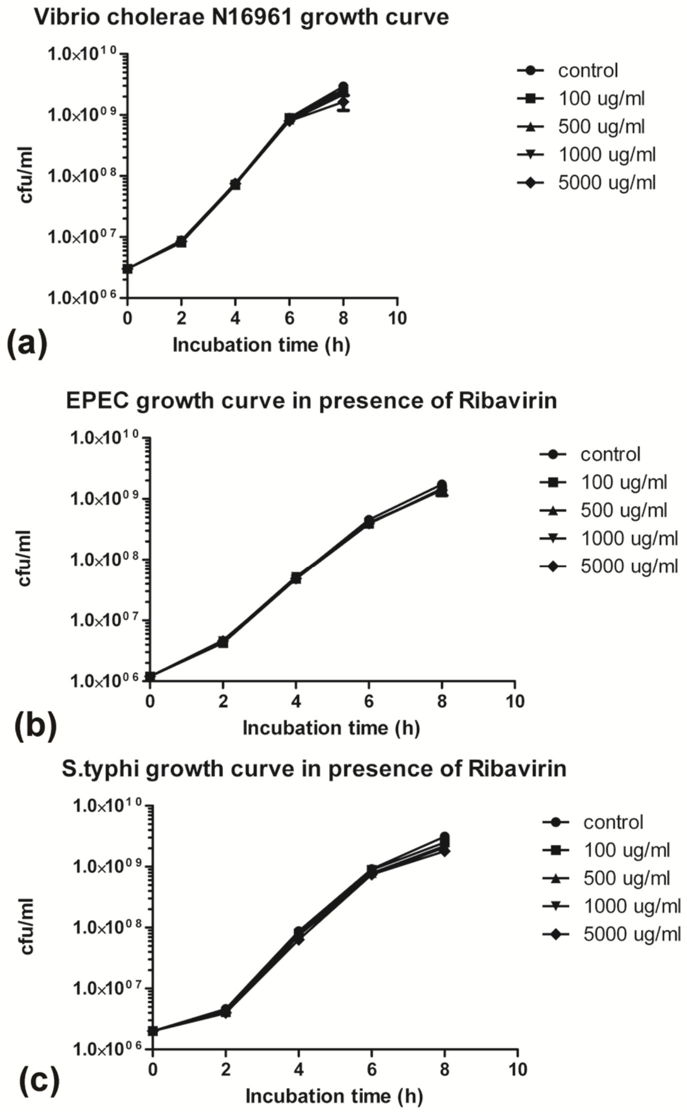

Supplementary figure S7. Ribavirin does not inhibit the growth of N16961$_{wt}$, S. Typhi Ty2 and EPEC strains *in vitro*: Bacterial strains, N16961 (a), EPEC (b) and S. Typhi Ty2 (c) were cultured in AKI media or LB at 37°C with shaking in the presence of increasing concentrations of ribavirin. Aliquots were collected at the indicated time-points and serial dilutions were plated on LB agar followed by colony counts.

Supplementary table-S1. All known crystal structures of LTTR in Protein Data Bank as on 15.08.2014

PDB ID	Title	Gene names	UniProtKB	Organism	PubMed ID
1AL3	COFACTOR BINDING FRAGMENT OF CYSB FROM KLEBSIELLA AEROGENES	cysB	P45600	Enterobacter aerogenes	9309218
3ONM	Effector binding Domain of LysR-Type transcription factor RovM from Y. pseudotuberculosis	RovM lrhA YpsIP31758_1452	A7FGQ3	Yersinia pseudotuberculosis IP 31758	21245528
4IHS	Crystal Structure of BenM_DBD/catB site 1 DNA Complex	benM benR ACIAD1435	O68014	Acinetobacter sp. ADP1	24100318
4IHT	Crystal Structure of BenM_DBD/benA site 1 DNA Complex	benM benR ACIAD1435	O68014	Acinetobacter sp. ADP1	24100318
2H99	Crystal structure of the effector binding domain of a BenM variant (R156H,T157S)	benM benR ACIAD1435	O68014	Acinetobacter sp. ADP1	19400783
2H9B	Crystal structure of the effector binding domain of a BenM variant (BenM R156H/T157S)	benM benR ACIAD1435	O68014	Acinetobacter sp. ADP1	19400783
2F97	Effector Binding Domain of BenM (crystals generated from high pH conditions)	benM benR ACIAD1435	O68014	Acinetobacter baylyi	17565172
2F8D	BenM effector-Binding domain crystallized from high pH conditions	benM benR ACIAD1435	O68014	Acinetobacter baylyi	17565172
2F6G	BenM effector binding domain	benM benR ACIAD1435	O68014	Acinetobacter baylyi	17291527
2F6P	BenM effector binding domain- SeMet derivative	benM benR ACIAD1435	O68014	Acinetobacter baylyi	17291527
2F78	BenM effector binding domain with its effector benzoate	benM benR ACIAD1435	O68014	Acinetobacter baylyi	17291527
2F7A	BenM effector binding domain with its effector, cis,cis-muconate	benM benR ACIAD1435	O68014	Acinetobacter baylyi	17291527
3GLB	Crystal structure of the effector binding domain of a CATM variant (R156H)	catM catR ACIAD1445	P07774	Acinetobacter sp. ADP1	19400783
2F7B	CatM effector binding domain	catM catR ACIAD1445	P07774	Acinetobacter baylyi	17291527
2F7C	CatM effector binding domain with its effector cis,cis-muconate	catM catR ACIAD1445	P07774	Acinetobacter baylyi	17291527
3KOS	Structure of the AmpR effector binding domain from Citrobacter freundii	ampR	P12529	Citrobacter freundii	20594961
3KOT	Structure of the Citrobacter freundii effector binding domain containing three amino acid substitutions: T103V, S221A and Y264F	ampR	P12529	Citrobacter freundii	20594961
3FXQ	Crystal structure of the LysR-type transcriptional regulator TsaR	tsaR	P94678	Comamonas testosteroni	20059681
3FXR	Crystal structure of TsaR in complex with sulfate	tsaR	P94678	Comamonas testosteroni	20059681
3FXU	Crystal structure of TsaR in complex with its effector p-toluenesulfonate	tsaR	P94678	Comamonas testosteroni	20059681
3FZJ	TsaR low resolution crystal structure, tetragonal form	tsaR	P94678	Comamonas testosteroni	20059681
3ISP	Crystal structure of ArgP from Mycobacterium tuberculosis	Rv1985c MTCY39.34	P9WMF5	Mycobacterium tuberculosis H37Rv	20036253
4JVC	Crystal structure of PqsR co-inducer binding domain	mvfR PA14_51340	Q02IG8	Pseudomonas aeruginosa UCBPP-PA14	23935486
4JVD	Crystal structure of PqsR coinducer binding domain of Pseudomonas aeruginosa with ligand NHQ	mvfR PA14_51340	Q02IG8	Pseudomonas aeruginosa UCBPP-PA14	23935486

4JVI	Crystal structure of PqsR co-inducer binding domain of Pseudomonas aeruginosa with inhibitor 3NH2-7Cl-C9QZN	mvfR PA14_51340	Q02IG8	Pseudomonas aeruginosa	23935486
2FYI	Crystal Structure of the Cofactor-Binding Domain of the Cbl Transcriptional Regulator	cbl b1987 JW1966	Q47083	Escherichia coli K-12	17010379
2Y7R	DNTR INDUCER BINDING DOMAIN	dntR	Q7WT50	Burkholderia sp. DNT	21692874
2Y7K	DNTR INDUCER BINDING DOMAIN IN COMPLEX WITH SALICYLATE. MONOCLINIC CRYSTAL FORM	dntR	Q7WT50	Burkholderia sp. DNT	21692874
2Y7P	DNTR INDUCER BINDING DOMAIN IN COMPLEX WITH SALICYLATE. TRIGONAL CRYSTAL FORM	dntR	Q7WT50	Burkholderia sp. DNT	21692874
2Y7W	DNTR INDUCER BINDING DOMAIN	dntR	Q7WT50	Burkholderia sp. DNT	21692874
2Y84	DNTR INDUCER BINDING DOMAIN	dntR	Q7WT50	Burkholderia sp. DNT	21692874
1UTB	DNTR FROM BURKHOLDERIA SP. STRAIN DNT	dntR	Q7WT50	Burkholderia sp. DNT	15210343
1UTH	DNTR FROM BURKHOLDERIA SP. STRAIN DNT IN COMPLEX WITH THIOCYANATE	dntR	Q7WT50	Burkholderia sp. DNT	15210343
4AB5	Regulatory domain structure of NMB2055 (MetR) a LysR family regulator from N. meningitidis	NMB2055	Q9JXG8	Neisseria meningitidis serogroup B	22750853
4AB6	Regulatory domain structure of NMB2055 (MetR), C103S C106S mutant, a LysR family regulator from N. meningitidis	NMB2055	Q9JXG8	Neisseria meningitidis serogroup B	22750853
3HHF	Structure of CrgA regulatory domain, a LysR-type transcriptional regulator from Neisseria meningitidis.	NMB1856	Q9JXW7	Neisseria meningitidis serogroup B	19474343
3HHG	Structure of CrgA, a LysR-type transcriptional regulator from Neisseria meningitidis.	NMB1856	Q9JXW7	Neisseria meningitidis serogroup B	19474343
3JV9	The structure of a reduced form of OxyR from N. meningitidis	NMB0173	Q9K1H8	Neisseria meningitidis MC58	20478059
3SZP	Full-length structure of the Vibrio cholerae virulence activator, AphB, a member of the LTTR protein family	VC_1049	Q9KT56	Vibrio cholerae	22053934
3T1B	Crystal structure of the full-length AphB N100E variant	VC_1049	Q9KT56	Vibrio cholerae	22053934
1IXC	Crystal structure of CbnR, a LysR family transcriptional regulator	cbnR	Q9WXC7	Cupriavidus necator	12706716
1IZ1	CRYSTAL STRUCTURE OF CBNR, A LYSR FAMILY TRANSCRIPTIONAL REGULATOR	cbnR	Q9WXC7	Cupriavidus necator	12706716
1YG2	Structure of the Vibrio cholerae virulence activator AphA	aphA	Q9X399	Vibrio cholerae	15647287

Selected structures for 3D and secondary structure alignment.

Supplementary table-S2. Unique LTTR present in Protein Data Bank

**Organism**	**PDB IDs of LTTR**
Enterobacter aerogenes	1AL3
Yersinia pseudotuberculosis IP 31758	3ONM
Acinetobacter baylyi	2F6G, 2F7B
Citrobacter freundii	3KOS
Comamonas testosteroni	3FXQ
Mycobacterium tuberculosis H37Rv	3ISP
Pseudomonas aeruginosa	4JVI
Escherichia coli K-12	2FYI
Burkholderia sp. DNT	1UTB
Neisseria meningitidis serogroup B	4AB5, 3HHF, 3JV9
Vibrio cholerae	3SZP
Cupriavidus necator	1IXC

Supplementary table-S3. Relative RMSD of 3SZP with other known LTTR crystal structures

**PDB ID**	**RMSD**	**Crystal resolution**
2F6G	0.773	1.91 Å
2FYI	1.607	2.8 Å
1AL3	1.759	1.8 Å
3ISP	0.735	2.7 Å
3JV9	2.673	2.39 Å
3FXQ	1.334	1.85 Å
4AB5	1.517	2.51 Å
2F7B	0.751	1.9 Å
1UTB	2.658	2.59 Å
1IXC	3.863	2.2 Å
4JVC	2.648	2.5 Å
3HHF	1.567	2.3 Å
3KOS	0.580	1.83 Å
3ONM	1.277	2.4 Å

Supplementary table-S4. Virtual screening result of FDA approved drug molecules with GScore threshold of -6 kcal/mol.

Sl. No.	DrugBank ID	Glide Gscore (kcal/mol)
1	DB01296	-8.122095
2	DB00584	-8.073006
3	DB08907	-8.034014
**4**	**DB00811**	**-7.945223**
5	DB00179	-7.730813
6	DB00141	-7.421428
7	DB01102	-7.29393
8	DB00521	-7.242351
9	DB08966	-7.207072
10	DB00194	-7.192936
11	DB01203	-7.100383
12	DB01017	-7.023042
13	DB06211	-6.994078
14	DB00879	-6.940989
15	DB09038	-6.909061
16	DB06213	-6.884019
17	DB06204	-6.848873

18	DB09082	-6.734487
19	DB00126	-6.684416
20	DB00938	-6.680155
21	DB11364	-6.594798
22	DB00927	-6.554131
23	DB00428	-6.532214
24	DB01325	-6.504317
25	DB00175	-6.502973
26	DB09150	-6.465774
27	DB00709	-6.380327
28	DB00249	-6.356197
29	DB00140	-6.332055
30	DB06207	-6.179346
31	DB11245	-6.178193
32	DB09221	-6.176051
33	DB08976	-6.170121
34	DB06794	-6.160143
35	DB01160	-6.149422
36	DB00322	-6.117411
37	DB01291	-6.112524

38	DB01001	-6.078053
39	DB09292	-6.059832
40	DB08819	-6.056807
41	DB01598	-6.054276
42	DB00198	-6.031316

Supplementary table-S5. Primers used in this study

**Primer Name**	**5'<-----Sequence----->3'**
AphB Full FP	GAGCTCCTAGATGACCTAAACCTC
AphB Full RP	CTCGAGTTATTGCAGGTGGTAGCCA
AphB mut FP	CGTATTTCTGCACCATCCGAACTGACAAAACGAATGATG
AphB mut RP	CATCATTCGTTTTGTCAGTTCGGATGGTGCAGAAATACG
AphB mut 5armFP	CACAAGATGTATGGTTTTC
AphB mut 5armRP	GTCAGAGTCAGTTTACGA
AphB mut 3armFP	CATGTTCAGAAGGTTTAG
AphB mut 3armRP	GTACGTACAACAGTAAGA
ctx A FP	TCGAGTTCATTTTGGGGTGCT
ctx A RP	CGGCGGTGCATGATGAATCC
tcpA FP	CGCAATTACAGTCGGTGGCT
tcpA RP	CACTTCCTGGTGCAATGGACT
tcpP FP	ACGTTGTTGATGAAGCTGACTGT
tcpP RP	GCCGGCTAATTCATGTTGATACC
toxT FP	CGTTGGGCAGATATTTGTGGT
toxT RP	TGAAACGCTAGCAAACCCAG
N16961 16srRNA FP	TAAACCACATGCTCCACCGC
N16961 16srRNA RP	CGTGGGGAGCAAACAGGATT
ler FP	CGAGAGCAGGAAGTTCAAAGTGT
ler RP	GTCTGCCCTTCTTCATTGCGG
escU FP	AGCTCTTGTCGTTGTTGCCT
escU RP	CGACGGCGCTCCCCTTTAAT
katG FP	CGTCGTTTGTTGTGGCCGAT
katG RP	TCGCCCCAGTTCACATCCAG
uvrA FP	ACTATCTCACGCTCTCCCGC
uvrA RP	ACCCAGCAGCCGTTCGTTAT
EPEC 16srRNA FP	TCGTCAGCTCGTGTTGTGAA
EPEC 16srRNA RP	CGCTTCTCTTTGTATGCGCC
Ty2 16srRNA FP	TGGGTTAAGTCCCGCAACG
Ty2 16srRNA RP	TGAGGTCCGCTTGCTCTCG
QseA full FP	GAATTCGTGTTTGCCAAAGTAGTTG
QseA full RP	CTCGAGTTACTTCTCTTTCCCGCG
Hrg full FP	GAATTCATGATAAAAACGGATCTCA
Hrg full RP	CTCGAGTTACTCTTCCACCATCCG
